# Investigating the molecular mechanisms of delirium-like neuropsychiatric disorder induced by electromagnetic pulse based on bioinformatics analysis

**DOI:** 10.1186/s13041-023-00998-z

**Published:** 2023-02-07

**Authors:** Xia-Jing Zhang, Zhi-Bin Xiao, Jun-Xiang Gu, Kun Chen, Jian Wang, Sheng-Long Xu, Ke-Ke Xing, Tao Chen

**Affiliations:** 1 Institute of Medical Research, Northwestern Polytechnical University, Xi’an, 710072 Shaanxi China; 2 Department of Human Anatomy, Histology and Embryology & K.K. Leung Brain Research Centre, The Fourth Military Medical University, No. 169 Changle West Road, Xi’an, 710032 China; 3 Department of Anesthesiology, The 986th Air Force Hospital, Xijing Hospital, The Fourth Military Medical University, Shaanxi 710032 Xi’an, China; 4 Department of Neurosurgery, The Second Affiliated Hospital of Xi’an Jiaotong University, Xi’an 710004, China; 5 Department of Neurosurgery, Tangdu Hospital, The Fourth Military Medical University, Xi’an, 710038 China; 6 Department of Radiation Biology, Faculty of Preventive Medicine, The Fourth Military Medical University, Xi’an, 710032 China

**Keywords:** Electromagnetic pulse, Neuronal injury, ceRNA, Bioinformatics analysis

## Abstract

Electromagnetic pulse (EMP), a unique type of electromagnetic radiation, may induce diverse neuropsychiatric disorders, such as irritability, hyperkinesis, retardation of learning and memory. However, the underlying mechanism of EMP exposure on neuronal injury has not been elucidated. Here, we aimed to delineate the regulatory expression networks based on high-throughput sequencing data to explore the possible molecular mechanisms related to EMP-induced delirium-like neuropsychiatric disorder in rats. It’s shown that EMP exposure induced anxiety, cognitive decline and short-term memory impairment. The expression profiles of the long noncoding RNAs (lncRNAs) and mRNAs, along with their biological function and regulatory network, were explored in rats after EMP exposure. We identified 41 differentially expressed lncRNAs (DELs) and 266 differentially expressed mRNAs (DEMs) between EMP and sham groups. Sixty-one co-expression relationships between 18 DELs and 56 DEMs were mostly associated with synapse- and metabolic-related pathways. We predicted 51 DEL-miRNA pairs and 290 miRNA-mRNA pairs using the miRanda database to constructed a DEL-miRNA-DEM network. LncRNA AABR07042999.1 and mRNA *Tph2*, *Slc6a4*, *Dbh* and *Th* were upregulated, and the contents of serotonin, dopamine and norepinephrine were increased in both PFC and HIP after EMP exposure. The current study provided a better understanding of the ceRNA network, which might reveal the pathological mechanism and provide more treatment options for the EMP-induced neurobehavioral disorder.

**Supplementary Information:**

The online version contains supplementary material available at 10.1186/s13041-023-00998-z.

## Introduction

Electromagnetic pulse (EMP) is a special kind of high power electromagnetic and is mainly used in military fields, which leads to irreversible damage to the electrical device such as wireless communication equipment, power supply system and military equipments [1]. Some studies report that EMP exposure causes deleterious effects in living organisms and induces human diseases [3]. Central nervous system is very sensitive to electromagnetic pulse radiation, it is suggested that a field strength of 400 kv/m EMP irradiation induces learning and memory impairment [4], and long-term exposure to 50 kv/m EMP causes a variety of irreversible cognitive decline leading to dementia [5]. However, the effect of EMP on delirium-like neuropsychiatric disorders and the underlying mechanism of neuronal injury has remained elusive.

Long non-coding RNA (lncRNA) is one of the most important members of non-coding RNA family with a length over 200 bp [6, 7]. LncRNAs are abundant in brain tissue and have been discovered to play a role in neuronal function and disease and is thus considered as biomarkers for diagnosis and prognosis of brain diseases [8–10]. However, the role and expression pattern of lncRNA in EMP induced nerve behavior disorder has not yet been clearly defined. Based on this, we used lncRNA sequencing to explore the potential mechanism of neuron injury caused by EMP.

In the present study, we found that EMP induced delirium-like neuropsychiatric disorders, such as anxiety, disturbed attention, disorientation and retardation of memory in rats. We identified the lncRNA and mRNA profiles of tissue samples from EMP radiated rats using lncRNA sequencing, GO annotation and KEGG pathways analyses. It’s indicated that the differentially expressed lncRNAs (DELs) and mRNAs (DEMs) were significantly enriched in serotonergic synapse, tryptophan metabolism, neurotransmitter biosynthetic process and neuromuscular process controlling balance. Further q-RT-PCR and ELISA detection confirmed that the increased levels of brain monoamine neurotransmitters such as serotonin (5-HT), dopamine (DA) and norepinephrine (NE) might underly the regulatory mechanism for EMP induced delirium-like neuropsychiatric disorders. Our study for the first time identifies the possible regulatory lncRNA and mRNA related to EMP induced delirium-like neuropsychiatric disorders in rats.

## Results

### EMP irradiation induces delirium-like neuropsychiatric disorder in rats

**Fig. 1:**
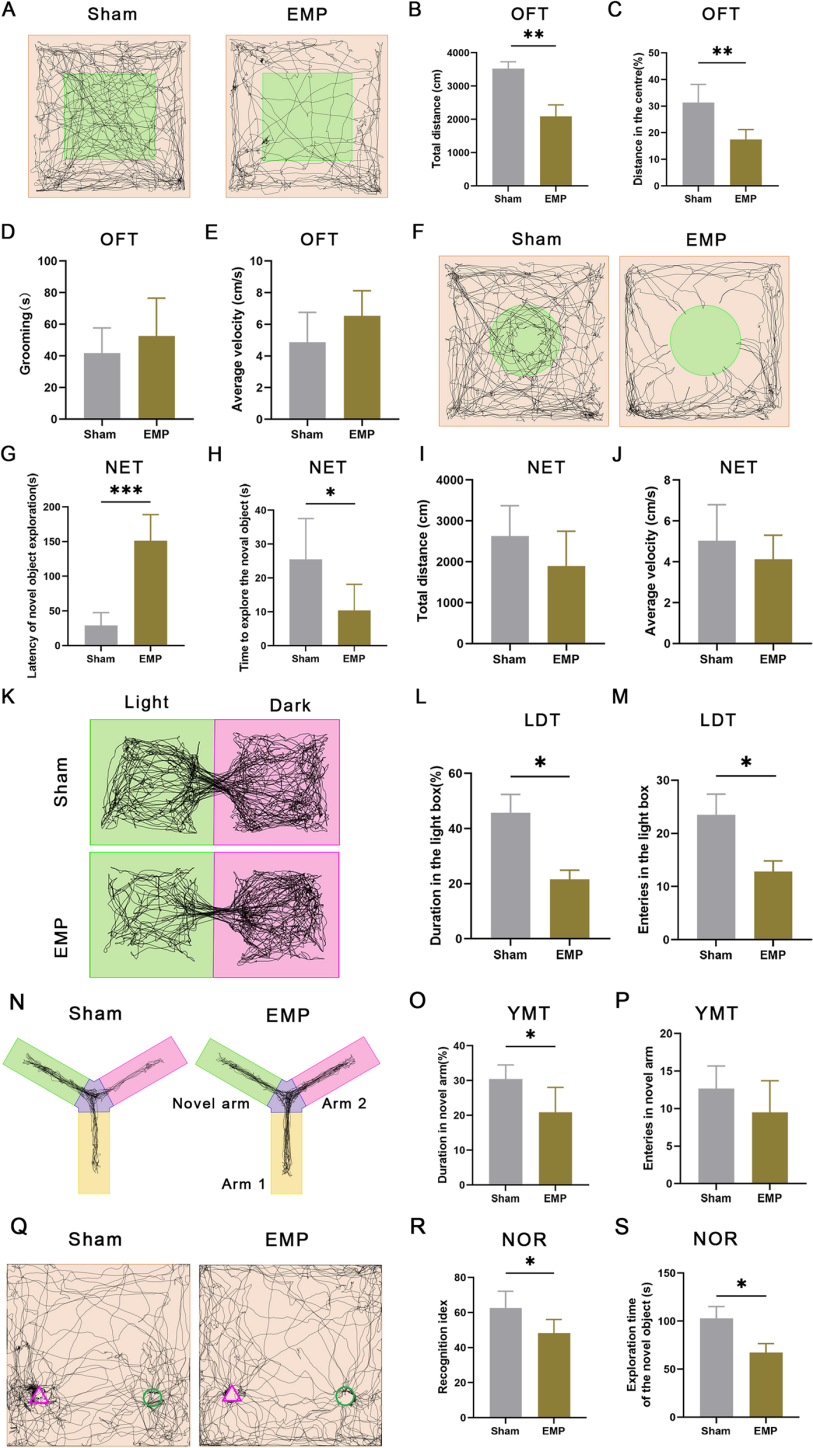
EMP irradiation induces delirium-like neuropsychiatric disorder in rats. **A** Representative images of movement tracks in open field test (OFT) for sham and EMP irradiated rats. **B**–**E** Total distance (**B**), percentage of distance in the center (**C**), Grooming time (**D**) and average velocity in OFT (**E**). ^*^p < 0.05, ^**^p < 0.01 (n = 6 rats in each group, unpaired t-test). **F** Trace plot of the novel object exploration test (NET) for sham and EMP irradiated rats. **G**–**J** Lantency (**G**), time to explore the novel object (**H**), total distance (**I**) and average velocity in NET (**J**). ^*^p < 0.05, ^***^p < 0.001 (n = 6 rats in each group, unpaired t-test). **K** The Trace images of the light/dark box test (LDT) for sham and EMP irradiated rats. **L**, **M** Duration (**L**) and entries (**M**) in the light box. ^*^p < 0.05 (n = 6 rats in each group, unpaired t-test). **N** Representative motion traces from the Y maze test (YMT) for sham and EMP irradiated rats. **O**, **P** Duration (**O**) and entries (**P**) in novel arm. ^*^p < 0.05 (n = 6 rats in each group, unpaired t-test). **Q** Trace plot of the novel object recognition test (NOR) for sham and EMP irradiated rats. **R**, **S** Recognition index (**R**) and time to explore the novel object (**S**). ^*^p < 0.05, ^***^p < 0.001 (n = 6 rats in each group, unpaired t-test)

We firstly assess whether EMP irradiation on rat will induce neuropsychiatric disorders. Delirium is a type of acute brain dysfunction characterized by consciousness disturbance, cognitive decline, short-term memory impairment, disturbed attention and disorientation [11]. We thus tested the emotional and cognitional functions by performing the open field test, novel objective recognition test, Y maze test and light/dark box test as reported before [12–14]. We observed that the percentage of cumulative duration and movement distance in the center area were significantly decreased after EMP exposure in the open field test (Fig. 1A–C), the latency of exploration was increased and exploration time was decreased in the EMP group compared to the sham group during the novel object exploration test (Fig. 1F–H). While the grooming time and average velocity in the open field test (Fig. 1D, E), total distance and average velocity in the novel objective exploration test (Fig. 1I, J) shows no statistically significance, indicating that the delirium-like behavior induced by EMP is not related to basic physical condition and locomotion of these animals. The light/dark box test showed that both the duration and entries into the light box were significantly decreased in the EMP group (Fig. 1L, M). The duration percentage in the novel arm of the Y-maze was decreased in the EMP group, while the entries into the novel arm showed no significant difference between sham and EMP group in the Y-maze test (Fig. 1O, P). The novel objective recognition test showed that both the recognition idex and exploration time of the novel object were significantly decreased in the EMP group (Fig. 1R, S). These behavioral test results revealed that EMP irradiation induced delirium-like neuropsychiatric disorders in rats.

### Identification of DELs and DEMs

**Fig. 2:**
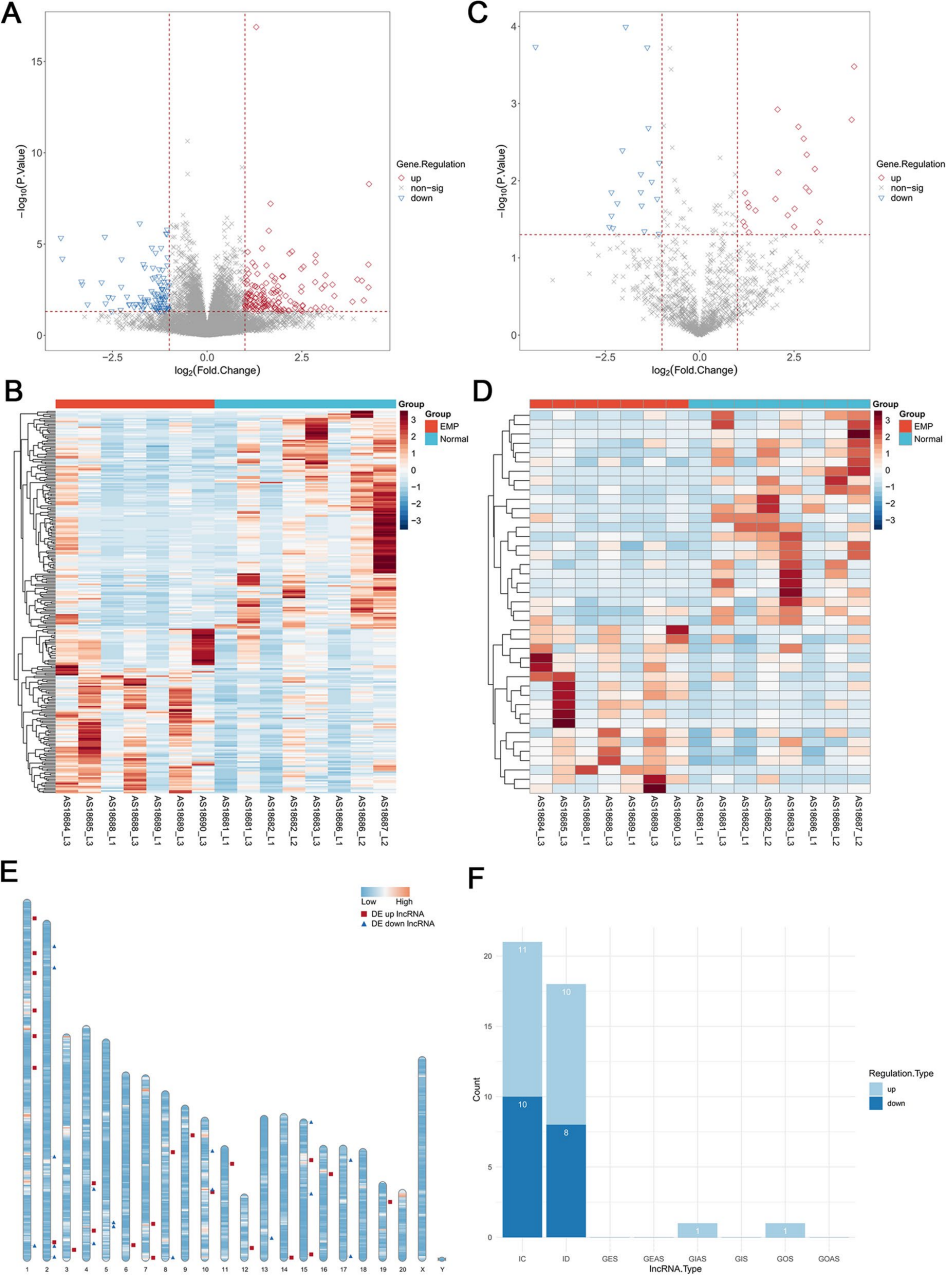
Identification of DELs and DEMs. **A**, **B** Volcano plot (**A**) and heatmap (**B**) of the mRNAs between rats in sham and EMP groups. Red and blue dots in (**A**) and lines in (**B**) indicate significant upregulated and downregulated genes, respectively. **C**, **D** Volcano plot (**C**) and heatmap (**D**) of the lncRNAs between rats in sham and EMP groups. Red and blue dots in (**C**) and lines in (**D**) indicate significant upregulated and downregulated genes, respectively. **E** The distribution of these identified DELs in all chromosomes. Chromosomes of rats are shown from left to right, with red squares for up-regulated differential lncRNAs and blue triangles for down-regulated differential lncRNAs. **F** The categorization of DELs: intergenic convergent (IC, 51%), intergenic divergent (ID, 44%), genetic intronic antisense (GIAS, 2.5%) and genetic overlapping same strand (GOS, 2.5%). DEMs, differentially expressed mRNAs, DELs, differentially expressed lncRNAs

We collected forebrain tissue from rats in EMP and sham group for RNA sequencing. After pre-processing the microarray data, DELs and DEMs between the EMP and sham groups were identified. As shown in Fig. 2A, B, 266 mRNAs were differentially expressed between the two groups, including 156 upregulated and 110 downregulated mRNAs. A total of 41 differentially expressed DELs were identified, with 23 upregulated and 18 downregulated DELs (Fig. 2C, D). The distribution of the identified DELs in all chromosomes is shown in Fig. 2E. To categorize the DELs, intergenic lncRNAs represented the largest category (95%) of all DELs, in which 51% were intergenic convergent and 44% were intergenic divergent. The remaining 5% of lncRNAs were genic, containing 2.5% that was genetic intronic antisense (GIAS) and 2.5% that was genetically overlapped with the same strand (GOS) (Fig. 2F). These results indicate DELs between the EMP and sham groups are mainly produced in the middle region of the two coding genes.

### GO and KEGG analysis of DEMs

**Fig. 3:**
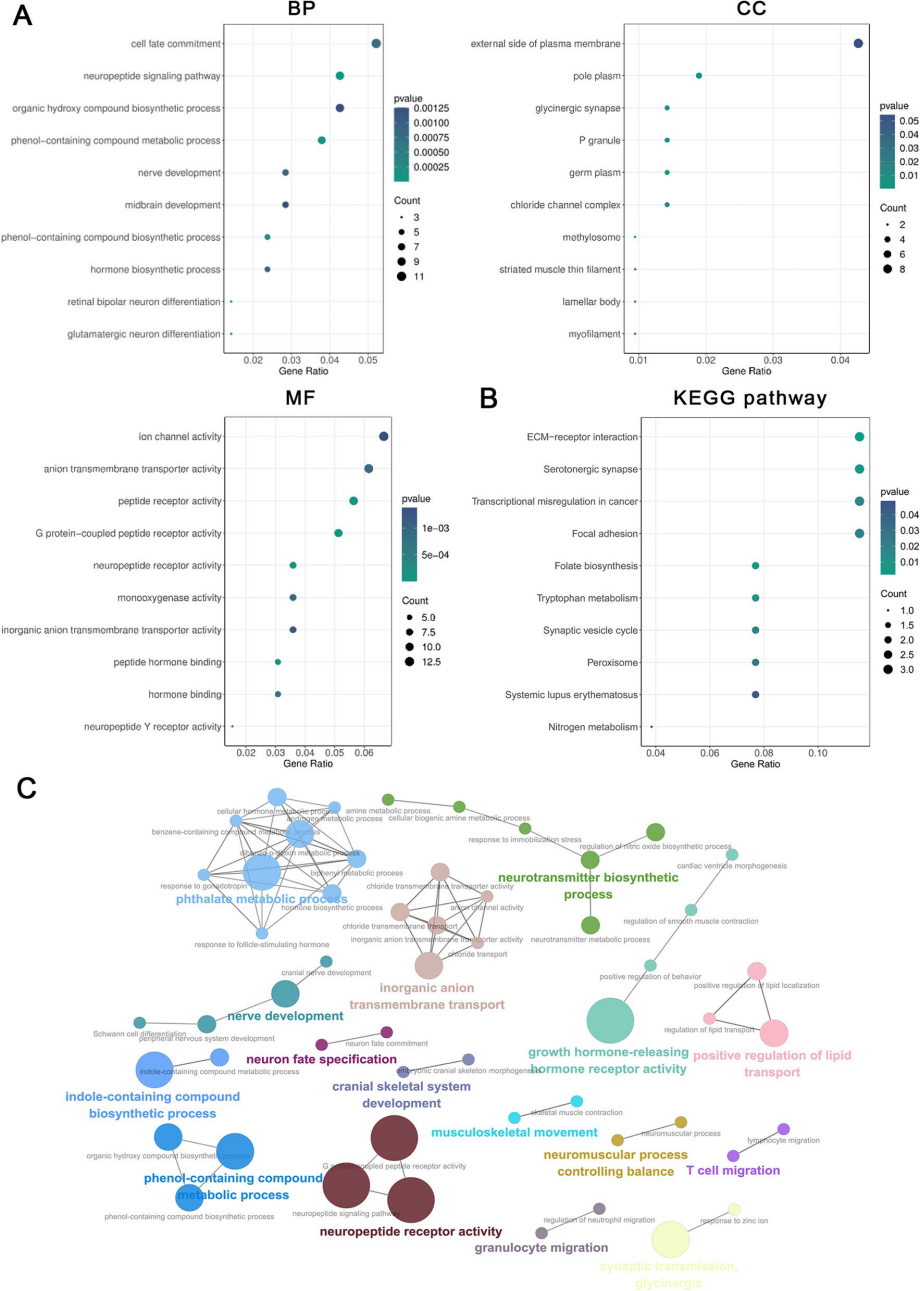
GO and KEGG analysis of DEMs. **A** Bubble plot of representative GO enrichment results of differentially expressed genes after EMP exposure. **B** Bubble plot of the enriched KEGG pathways. **C** Enriched GO network groups using ClueGO. DEMs, differentially expressed mRNAs; GO, Gene ontology; ClueGO, a functional plug-in in Cytoscape that annotates ontology of DEMs

To obtain a primary understanding of the biological functions and pathways of the DEMs, gene ontology (GO) annotation and Kyoto Encyclopedia of Genes and Genomes (KEGG) pathway analyses were conducted. For biological process (BP), the DEMs were mostly associated with nerve development, glutamatergic neuron differentiation, phenol-containing compound biosynthetic processes and phenol-containing compound metabolic processes. The functions of these DEMs in the cellular component (CC) mainly concentrated on the external side of the plasma membrane, chloride channel complex and glycinergic synapse. The main molecular functions (MF) that were significantly enriched in DEMs were ion channel activity, G protein-coupled peptide receptor activity, organic hydroxy compound biosynthetic process, phenol-containing compound biosynthetic process and phenol-containing compound metabolic process (Fig. 3A). KEGG pathway analysis demonstrated that these DEMs were significantly enriched in the serotonergic synapse, tryptophan metabolism and the synaptic vesicle cycle (Fig. 3B). ClueGO, a functional plug-in in Cytoscape that annotates gene ontology, indicated that the DEMs were mainly associated with the biological processes of nerves, including nerve development, neuron fate specification, neurotransmitter biosynthetic process and neuromuscular process controlling balance (Fig. 3C). Together, these results suggest that EMP induced delirium-like neuropsychiatric disorders may be related to the synthesis, metabolism and reuptake of neurotransmitters in rats’ brain.

**Fig. 4:**
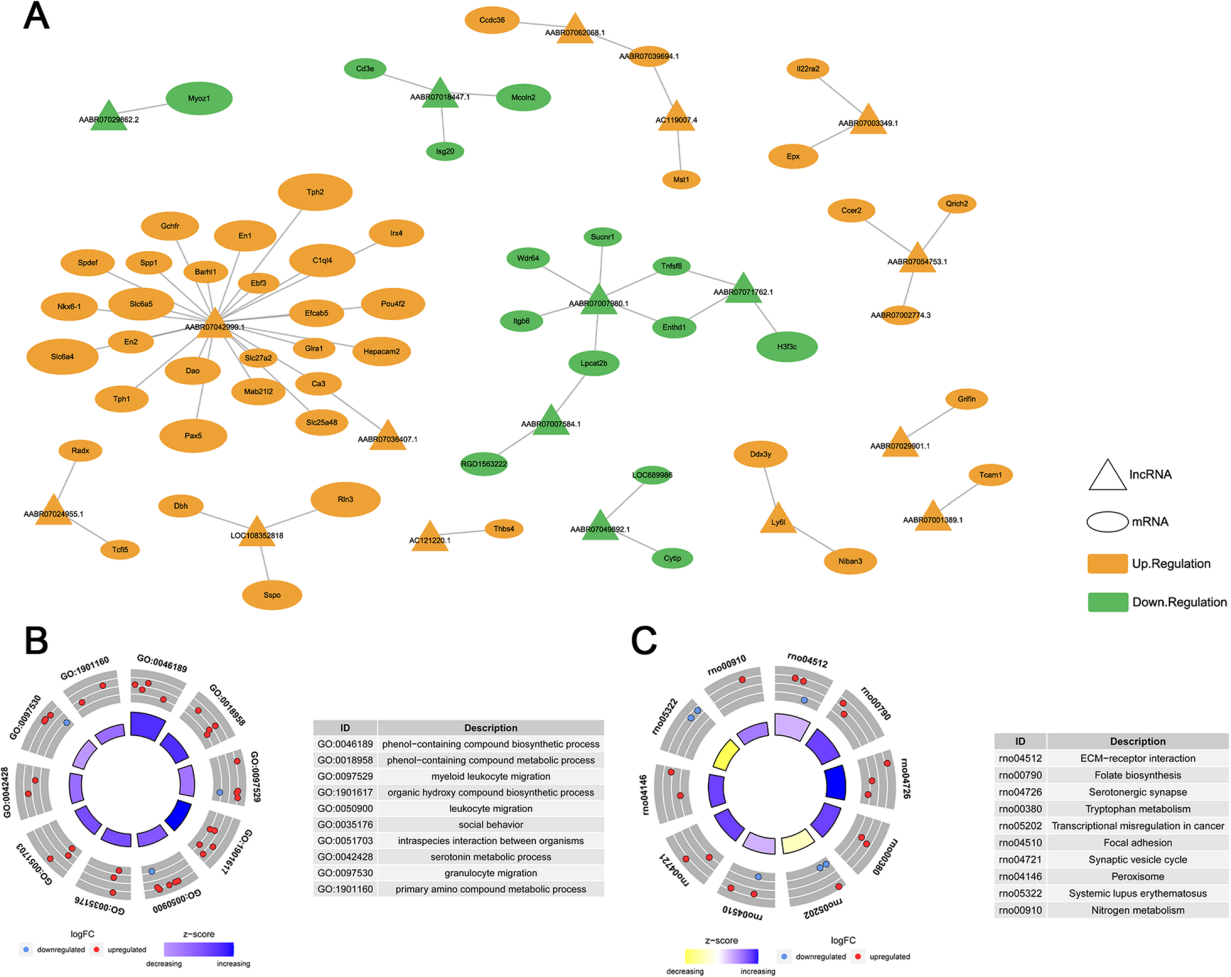
Construction of DEL-DEM co-expression network. **A** DEL-DEM co-expression network. Oval represents mRNA while triangle indicates lncRNA. The larger size of oval indicates the stronger correlation. **B** GO biological process enrichment analysis of the co-expressed mRNAs. **C** KEGG pathway enrichment analysis of the co-expressed mRNAs

The prediction of the biological function of lncRNAs was based on annotations of the corresponding mRNAs functions. To construct a differentially expressed lncRNA-mRNA co-expression network, we calculated the correlation between DELs and DEMs using Pearson analysis. Our results revealed 61 differentially expressed lncRNA-mRNA pairs, including 18 DELs and 56 DEMs (Fig. 4A, Additional file 3: Table S3). Co-expressed DEMs were mostly enriched in synapse- and metabolic-related pathways, such as serotonin metabolic process, serotonergic synapse, tryptophan metabolism, ECM-receptor interaction and primary amino compound metabolic processes (Fig. 4B, C).

### The regulatory mechanisms of DELs

**Fig. 5:**
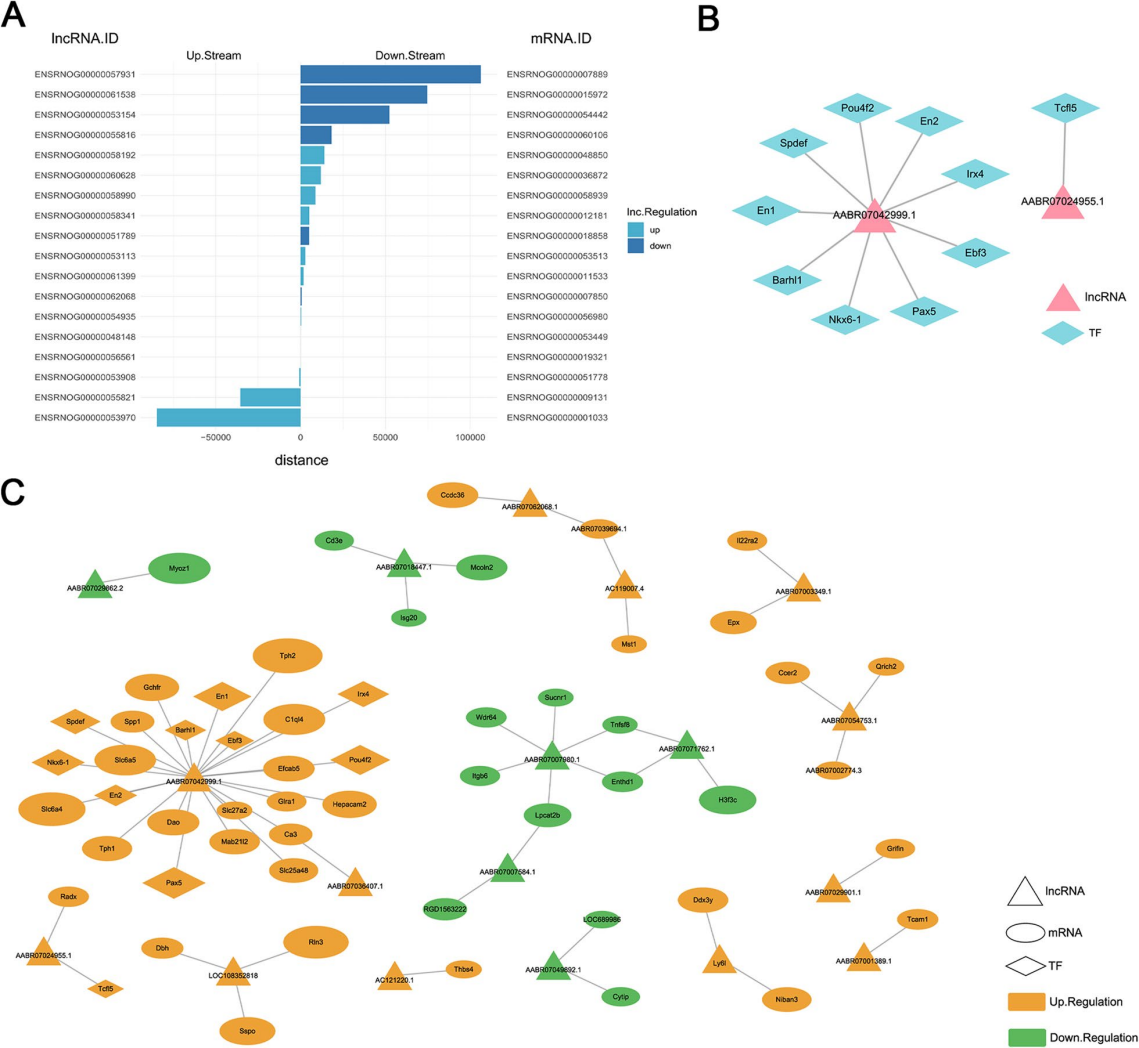
The regulatory mechanisms of DELs. **A** The *cis*-Regulatory relationship between lncRNA and protein-coding genes. Y-axis represents lncRNA position in chromosome. X-axis represents *cis*-Regulatory DEL and DEM pairs. **B** DEL-TF network. The rhomboid represents the transcription factor. The triangle represents the lncRNA. **C** DEL-TF-mRNA regulatory network. The combination of DEL-TF network and DEL-DEM co-expression network. The oval represents the mRNA, with up-regulated genes marked with yellow and down-regulated genes marked with green

Next, we determined the *cis*- and *trans*-regulatory roles of DELs in gene expression based on the interaction between DELs and DEMs. Gene transcripts within 300 kb upstream or downstream of the DELs on the same chromosome were considered *cis*-regulated genes. A total of 18 DELs were identified to regulate their corresponding nearby coding genes in a *cis*-manner (Fig. 5A). In addition, lncRNAs can modulate target gene expression by interacting with transcription factors (TFs). We identified 10 TFs and established a differentially expressed DEL-TF network, of which AABR07042999.1 corresponded to nine TFs (Fig. 5B). Furthermore, the isolated mRNAs in the co-expression network were used to establish the DEL-TF-mRNA regulatory network (Fig. 5C).

### Construction of ceRNA network

**Fig. 6:**
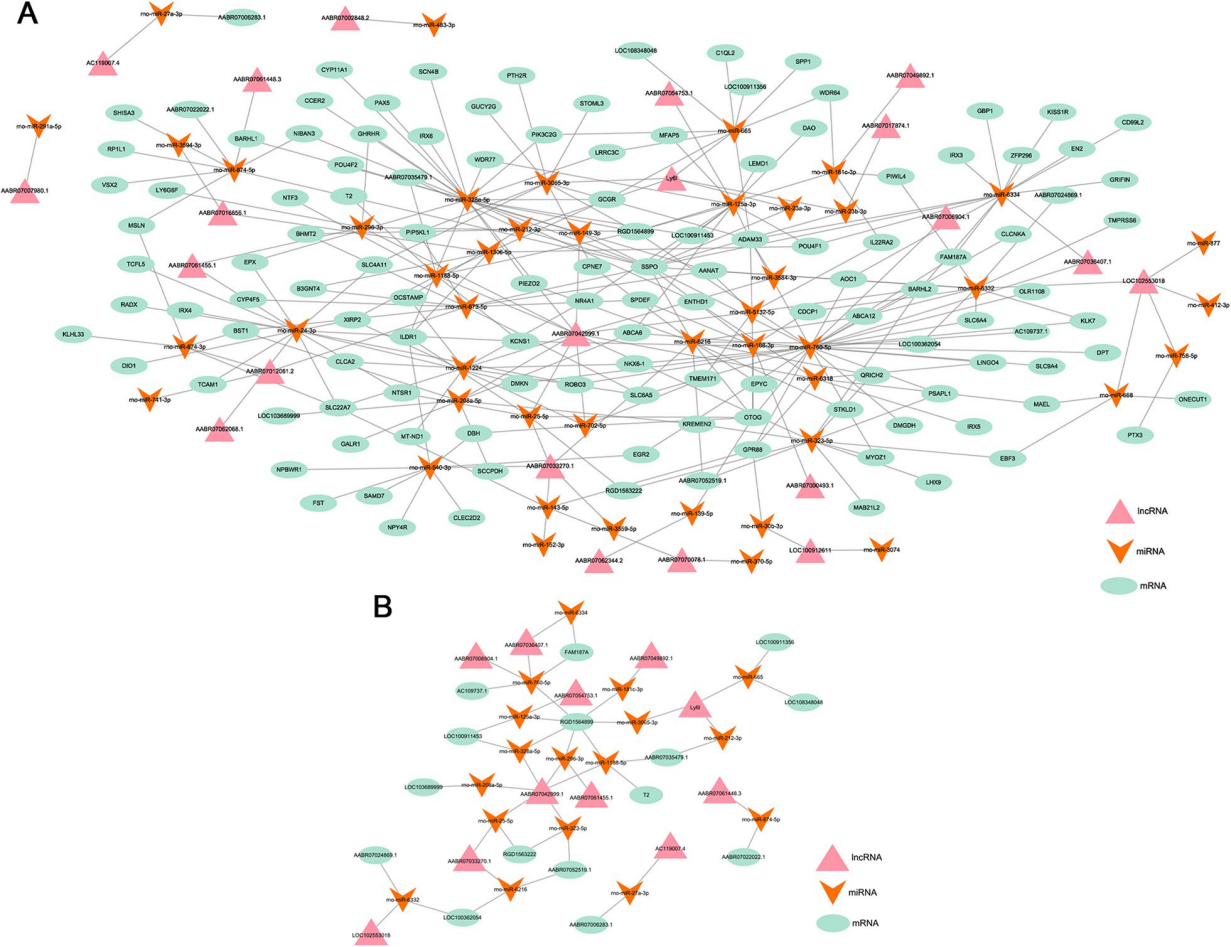
Construction of ceRNA network. **A** CeRNA interaction network of DELs-miRNA-mRNA. **B** CeRNA interaction network of DEL-miRNA-DEM. The triangle represents the lncRNA, the arrows represents the miRNA and the oval represents the mRNA

According to the ceRNA hypothesis, ceRNAs act as miRNA sponges to indirectly regulate gene expression. To further explore the regulatory role of DELs in neurons following EMP stimulation, 51 differentially expressed lncRNA-miRNA pairs and 290 miRNA-mRNA pairs were predicted using the miRanda online tool to construct a DEL-miRNA-mRNA network (Fig. 6A). The DEL-miRNA-DEM network was constructed by overlapping the mRNA in the ceRNA network and the DEMs between the EMP and sham groups (Fig. 6B). LncRNAs such as AABR07042999.1, AABR07061455.1 and AABR07033270.1 can form ceRNA interaction pairs with several different miRNAs and mRNAs. These findings provide new insights into the mechanism of EMP-induced neurobehavioral disorders.

### Validation of DELs and DEMs by qRT-PCR

**Fig. 7:**
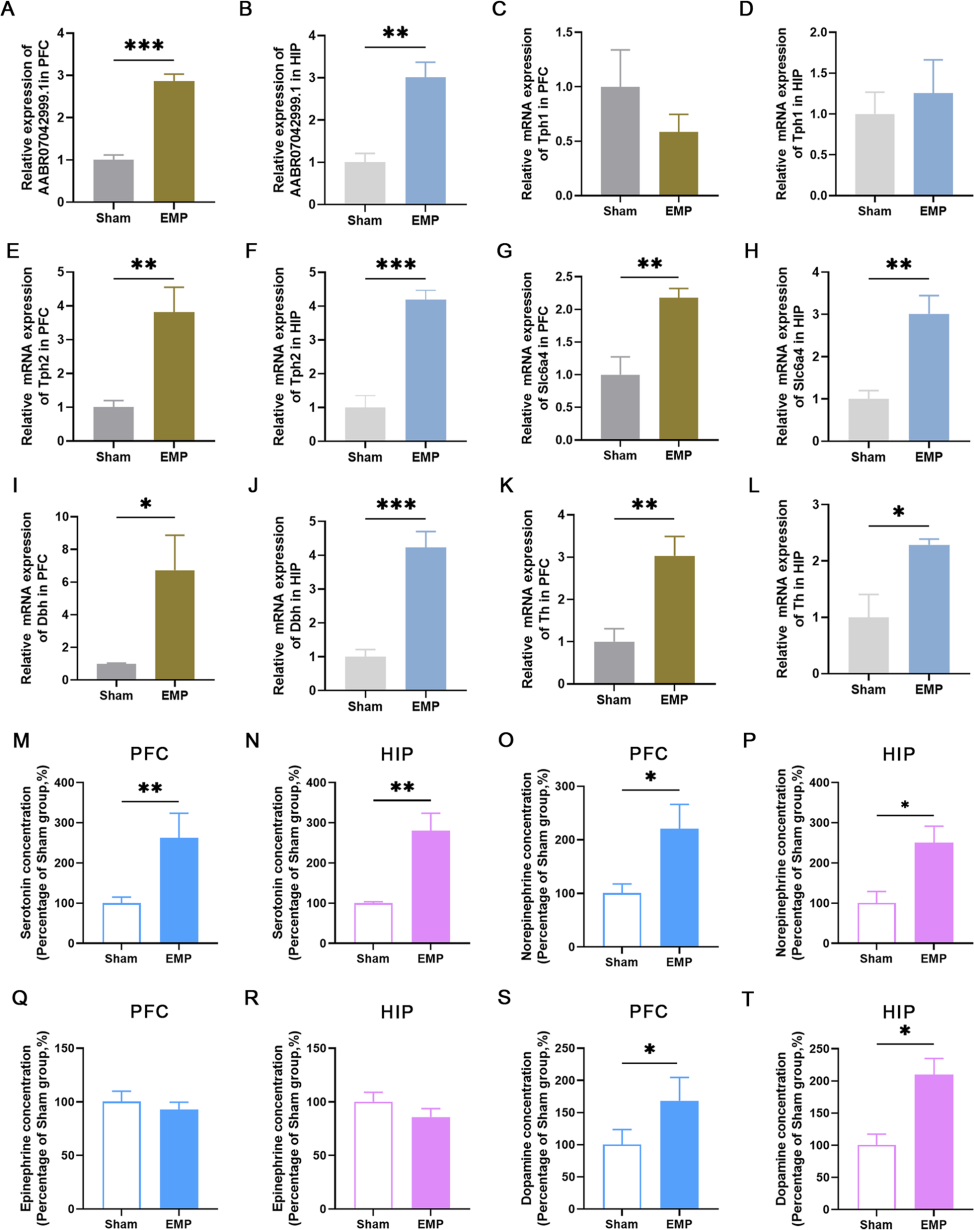
QRT-PCR validation and neurotransmitter determination in the prefrontal cortex (PFC) and hippocampus (HIP). **A**, **B** LncRNA AABR07042999.1 were upregulated in the PFC and HIP. **C**, **D** Tph1 shows no significant difference in the PFC and HIP. Tph2 (**E**, **F**), Slc6a4 (**G**, **H**), Dbh (**I**, **J**) and Th (**K**, **L**) were upregulated in both PFC and HIP after EMP exposure. The concentrations of 5-HT (**M**, **N**), NE (**O**–**P**) and DA (**S**, **T**) were increased in both PFC and HIP. There is no significant difference in epinephrine concentration between the two groups (**Q** and** R**). ^*^p < 0.05, ^**^p < 0.01, ^***^p < 0.001 (n = 6 mice in each group, unpaired t-test)

Delirium is a syndrome characterized by diffuse and temporary brain damage and disfunction [15]. In rodents, the main cognitive and learning deficits of delirium-like neuropsychiatric disorders are associated with the dysfunction in the prefrontal cortex (PFC) or hippocampus (HIP). Having observed profound transcriptome changes in the forebrain tissues of the EMP irradiation-exposed animals, we thus validated the reliability of the RNA sequencing selected by differentially expression analysis (Fig. 2), by qRT-PCR in the PFC and HIP. In the EMP group, lncRNA AABR07042999.1 and co-expressed mRNA *Tph2, Slc6a4, Dbh*, and *Th* were upregulated in both the PFC and HIP after EMP exposure when compared to the sham control (Fig. 7A–L).

### EMP-induced neurotransmitter changes in the PFC and hippocampus

We also confirmed that aberrant gene expression changes were associated with neurotransmitter changes in the PFC and HIP. ELISA showed that the concentrations of 5-HT, NE, and DA were increased in both the PFC (Fig. 7M, O and S), and HIP (Fig. 7N, P and T) after EMP exposure, while there was no significant difference in epinephrine concentration between EMP and sham groups (Fig. 7Q and  R).

## Discussion

The degree of EMP-induced health hazards is correlated to the intensity of the radiation source and irradiation time. The brain is vulnerable to EMP radiation, which often results in abnormal behavior and impaired cognitive function. There is evidence that long-term exposure to EMP (50 kV/m, 100 pulses, 12–18 months) leads to cognitive decline in rats by increasing Aβ accumulation in the brain [5]. Repeated EMP exposure (400 kV/m, 200 pulses, 3 days) causes a series of biological effects such as microglial activation, neuroinflammation, neuronal apoptosis, and cognitive impairment [4]. In this study, we found that a single exposure to EMP (700 kV/m, 400 pulses) induced delirium-like neuropsychiatric disorders. Our objective was to mimic the brain function and behavioral alterations in animals after a momentary exposure to the EMP environment and investigate the possible molecular mechanism for the neuropsychiatric effects using RNA sequencing combined with bioinformatics analysis.

Delirium may be the result of dysfunction of multiple interacting neurotransmitter systems. Changes in the levels of various amino acids being precursors of cerebral neurotransmitters may affect their function and, thus, contribute to the development of delirium. In the present study, lncRNA sequencing was used to investigate changes in mRNA and lncRNA expression after EMP radiation. Bioinformatics analysis determined 266 DEMs and 41 DELs. Further GO functional enrichment analysis and KEGG pathway analysis performed on DEMs revealed serotonergic synapses, tryptophan metabolism, neurotransmitter biosynthetic processes, neuromuscular process controlling balance related to the rat nervous system. This is consistent with previous reports that neurotransmitter disorders play a crucial role in the development of delirium, especially the dopamine, acetylcholine, and serotonergic systems [16]. Here, we aimed to understand the regulatory mechanisms and functions of DELs in rats. However, most rats’ lncRNAs were not functionally annotated and had rarely been studied. Therefore, we constructed a co-expression network between DELs and DEMs. The regulatory function of lncRNAs was indirectly predicted by mRNA co-expression with lncRNAs. Co-expressed DEMs were mostly enriched in synapse- and metabolism-related pathways, such as serotonin metabolic processes, serotonergic synapses, and tryptophan metabolism.

LncRNAs can function in *cis*- or *trans*-configurations of gene coding [17]. Eighteen DELs were identified as transcriptional regulators of nearby coding genes in a *cis*-configuration. For *trans*-configurations, the lncRNA AABR07042999.1 was found to modify nine TFs. In the lncRNA-TF-mRNA co-expression network, we only identified that lncRNA AABR07042999.1 - *En2* - *Slc6a4* might participate in EMP induced neurotransmitter disorders*.* Since lncRNA may act as a ceRNA to sponge miRNA and indirectly regulate gene expression [18], we also constructed a DEL-miRNA-DEM network analysis. LncRNA AABR07042999.1 and its co-expressing coding genes *Tph1*, *Tph2*, and *Slc6a4* were involved in the synthesis and reuptake of 5-HT, and *Th* and *Dbh* encoding key enzymes in the production of DA and NE. Monoamine transmitters represented by 5-HT, DA and NE are the main neurotransmitters involved in mental and emotional activities. 5-HT is one of the neurotransmitters that may play an important role in medical and surgical delirium. Normal serotonin synthesis and release in the human brain is, among others, dependent on the availability of its precursor tryptophan (Trp) from blood. Both increased and decreased serotonergic activity have been associated with delirium [19, 20]. This is in consistent with the KEGG analysis that DEMs after EMP radiation were enriched in Trp metabolism. DA may play an important role in the pathophysiological mechanism of delirium. Clinical application of levodopa promotes the occurrence of delirium [21] and haloperidol, a DA receptor inhibitor, effectively improves the psychiatric symptoms of delirium [22]. Therefore, we speculate that the lncRNA AABR07042999.1 might act as a ceRNA to sponge miRNA and indirectly regulate *Tph2*, *Slc6a4*, *Dbh*, and *Th* expression in the EMP-induced delirium-like neuropsychiatric disorders.

In summary, a series of bioinformatic analyses, including differential expression, chromosomal localization, classification, and functional enrichment of lncRNA and mRNA in rats, were conducted to explore the changes of lncRNA in forebrain after EMP exposure in rats. The lncRNA co-expression and ceRNA networks were constructed to explore the potential regulatory mechanisms for lncRNAs in EMP associated neurotransmitter disorders and delirium-like neurobehavioral changes. However, other than the PFC and hippocampus, other brain regions such as the amygdala and striatum are also important in delirium-like neuropsychiatric disorders [16] and qPCR and ELISA confirmation will be carried out in amygdala and striatum. Future functional experiments will also be needed to provide a complete picture of the neuropsychiatric effects of EMP exposure on the brain.

## Methods and materials

### Animals

Male Sprague–Dawley (SD) rats weighing 200 ~ 250 g, with free access to food and water, were purchased from the Experimental Animal Centre of the Air Force Military Medical University (Xi'an, China). The animals were housed in a temperature-controlled room and exposed to a 12-h light/dark cycle, then acclimated to the environment for 7 days before the experiments. The experimental protocol was approved by the Ethics Committee for Animal Experimentation.

### EMP exposure system

As previously described, an all-solid-state nanosecond generator was used [23]. Briefly, an electromagnetic pulse was executed through a spark gap generator and transmitted into a gigahertz transverse electromagnetic cell. This setup delivered peak intensity 700 kV/m, 1 Hz electromagnetic pulse, and the total pulse repetition was 400. A Tektronix 7000B oscilloscope (Tektronix, Beaverton, OR) was used to observe the pulse waveform. During the experiment, all SD rats were kept in transparent plastic boxes measuring 20 cm × 8 cm × 8 cm. EMP group animals were totally exposed to EMP radiation while sham group rats were placed in the EMP exposure space but not radiated. The body temperature measurement before and after EMP exposed showed no significant changes.

### Open field test

As previously described, we use open field test measures hyperactivity through locomotion and anxious behavior [24]. The apparatus was a black plastic open arena without any bedding (40 cm × 40 cm × 30 cm, L × W × H), which was placed under diffuse even lighting (30 lx). A center zone (17.5 cm L × 17.5 cm W) was identified and marked using Tracking Master V3.0 software (Shanghai Vanbi Intelligent Technology Co., Ltd.) and a camera directly overhead connected to Tracking Master V3.0 software tracked the movement of the animals. Rats were placed in the center of the box and allowed to explore the arena for 10 min, and the dependent measures were: total time spent in the center divided by total exploration time and distance traveled in the center divided by total distance traveled. The apparatus was cleaned with 70% ethanol after each rat was tested.

### Y maze test

It has been demonstrated that the Y-maze test can be a reliable, noninvasive way to assess cognitive changes in rats through the measurement of their spontaneous alternation behavior [25]. The maze used in the our study consisted of three arms (35 cm × 10 cm × 25 cm, L × W × H) and an equilateral triangular central area. Rats were placed at the end of one arm and allowed to explore only two arms of the maze during a 10 min period. They were not permitted to explore the third arm (novel arm) of the maze. Arm entry was quantified when an animal had its hind paws completely within a maze arm. The novel arm entries and the duration were recorded. The maze was carefully cleaned with 70% ethanol between each rat was tested to minimize odor cues.

### Novel object recognition test

Novel object recognition test was conducted in an open-field arena. Briefly, the animals were placed in the field with two identical objects positioned at an equal distance. On the consecutive day, the rats were allowed to explore the open field in the presence of a familiar object, and a novel object to test recognition memory. The time spent exploring each object and the recognition index percentage were recorded [26].

$$\mathrm{recognition\, index }=\frac{\mathrm{time \,spent\, studying\, the\, novel \,object}}{\mathrm{time\, spent\, studying\, the\, novel \,object}+\mathrm{time \,spent \,studying \,the\, familiar \,object} }\times 100\mathrm{\%}$$

### Novel object exploration test

Novel object exploration test was performed as previously described [27]. Briefly, Rats were placed in the center of the open field arena, and allowed to explore the arena 30 min for training, then returned to homecage for 5 min, during which time a novel object was placed in the center of the open field. The rat was then placed back into the chamber and allowed to explore the test arena containing the novel object for 10 min while video recording. Latency of novel object exploration and the times of novel object exploration were recorded for further analysis.

### Light/dark box test

The light/dark box is another test to assess rats’ explorative behavior and anxiety [28]. The light–dark box (32 cm × 40 cm × 30 cm, L × W × H) consists of two equal chambers: a white walled light chamber and a black walled dark chamber. There is an opening within the box at the base for the exploratory behaviour. To begin each test session, a rat was placed in the light area for 5 min and allowed to move between both the compartments inside the apparatus, a digital camera mounted directly above the apparatus recorded the total time spent in light and dark section with transition between two sections during the test session (Tracking Master V3.0 software (Shanghai Vanbi Intelligent Technology Co., Ltd.)). The box was cleaned with 70% ethanol at the end of the recording sequence for each rat.

### Data resource and processing

In EMP group, only rats that presented with delirium-like behavior were selected for RNA-sequencing then subjected to bioinformatics analysis. For forebrain tissue collection, rats were immediately decapitated after the behaviour tests with minimizing suffering procedures. Total RNA was extracted with Trizol reagent (Invitrogen, Carlsbad, CA, USA). Amount of 3 μg total RNA for each sample was used as input material for the RNA sequencing sample preparations. RNA sequencing libraries were generated using the NEBNext Ultra Directional RNA Library Prep Kit (Illumina) from total RNA.

Quality assessment and preprocessing of raw sequencing data were essential for the high-level analysis of the subsequent study. In our study, FastQC software (http://www.bioinformatics.bbsrc.ac.uk/projects/fastqc) was utilized to check the quality of raw sequencing data. Then, the low-quality sequencing data and adapters were excluded by the Trimmomatic software. Afterward, the STAR tool was used to map the alignment of reads with the Rat reference genome (Rnor_6.0 (GCA_000001895.4). Finally, the HTSeq-count was performed to calculating the raw read-count and calculated the expression abundance of lncRNAs and mRNAs.

### Differentially expression analysis

After processing the sequencing data, DESeq2 package in R was applied for the differentially expressed analysis. The lncRNAs and mRNAs met the selection criteria of *P* < 0.05 and |log_2_(Fold Change)|> 1 were considered as significantly differentially expressed between the EMP and sham groups (Additional file 1: Table S1, Additional file 2: Table S2).

### DELs categorization

Based on modifications of the previous classification [29], we classified rat lncRNAs according to their gene positions related to the most proximal protein-coding genes. The lncRNA genes were first considered as intergenic and genic according to whether they intersect a protein-coding gene. Intergenic lncRNAs were further categorized as convergent (IC) and divergent (ID) depended on their transcribed from the same or opposite strand. Genic lncRNAs were further classified into genic exonic (genic exonic same strand (GES) and genic exonic antisense (GEAS)), genic intronic (genic intronic same strand (GIS) and genic intronic antisense (GIAS)), or overlapping (genic overlapping same strand (GOS) and genic overlapping antisense (GOAS)) based on they overlapped with the exons or introns of a protein-coding gene.

### DEL-DEM co-expression analyses

For co-expression analyses of identified DELs and DEMs, the Pearson correlation analysis was performed to assess the relationship between the expression level of DELs and DEMs. The lncRNA-mRNA co-expression network was visualized by the Cytoscape software. |Pearson Correlation Coefficient (PCC)|> 0.9 and *P*-value < 0.05 were was set to indicate a co-expression correlation.

### Functional enrichment analysis

To explore the latent biological functions and pathways of the DEMs between EMP and normal groups and mRNAs identified from the co-expression network mentioned above, GO annotation and KEGG pathway analyses were conducted with the R package clusterProfler. Besides, DEMs were further submitted into the ClueGO, a plug-in Cytoscape, to establish a function network of GO enrichment analysis. *P* < 0.05 was regarded as the cut-off criterion.

### *Cis*- and *trans*-regulated analysis of DELs

Previous studies suggested that lncRNAs regulated transcription of their nearby genes by acting in *cis*- and *trans*-manners. For cis prediction, the genes located on the same chromosome within a 300 kb region upstream or downstream of lncRNAs were identified as *cis*-regulated genes. For trans prediction, we focused on that lncRNAs might regulate the expression levels of transcription factors (TFs) via the trans manner. Hence, the mRNAs co-expressed with lncRNAs were interacted with TFs obtained from the AnimalTFDB database to identify the trans-regulated genes.

### Construction of the ceRNA co-expression network

We used the miRanda database to predict the binding between these miRNAs-lncRNAs/miRNAs using the threshold of score ≥ 150 and energy ≤ -25 kcal/mol. Then, the Cytoscape software was applied to construct a lncRNA-miRNA-mRNA network based on the lncRNA-miRNA pairs and miRNA-mRNA pairs.

### RT-qPCR validation

Because delirium is a neurobehavioral syndrome caused by a transient disruption of normal neuronal activity mediated by alteration in neurotransmitter and neuronal network function [30], we chosen several neurotransmitter related DE mRNAs, and their co-expressed DE lncRNAs for validation of the RNA-sequencing results in EMP versus sham rats using qRT-PCR. In brief, total RNA was extracted from liquid nitrogen-frozen prefrontal cortex and hippocampus tissues. Each reverse transcription reaction consisted of 0.5 μg RNA, 2 μl of 5 × TransScript All-in-one SuperMix for qPCR and 0.5 μl of gDNA Remover, in a total volume of 10 μl. Reactions were performed in a GeneAmp® PCR System 9700 (Applied Biosystems, USA) for 15 min.

Real-time PCR was performed using LightCycler® 480 II Real-time PCR Instrument (Roche, Swiss) and then reactions were incubated in a 384-well optical plate (Roche, Swiss) at 94℃ for 30 s, followed by 45 cycles of 94℃ for 5 s, 60℃ for 30 s. Each sample was run in triplicate for analysis. The primer sequences were designed in the laboratory and synthesized by TsingKe Biotech based on the mRNA sequences obtained from the NCBI database are shown in Table 1. The expression levels of mRNAs were normalized to (GAPDH, ACTB) and were calculated using the 2^−ΔΔCt^ method [31].

**Table 1:** Primer sequences of changed lncRNAs and four mRNAs

Gene symbol	GeneBank	Forward primer	Reverse primer
AABR07042999.1	ENSRNOG 00000058341	CTGCAAGTGGTGACAGATG	TGACCAAGGGAGGGAAGA
Tph1	ENSRNOG 00000011672	CATATTGAGTCCCGGAAATCG	AGCTGTTCTCGGTTGATG
Tph2	ENSRNOG 00000003880	TTTCTGACAAGGCGTGTGTAA	TAAGCATCTTGGAAGGTGGT
Slc6a4	ENSRNOG 00000003476	ACACGGTCCTAACCACTAT	GTGTCACCATCGAATGTGGTA
Dbh	ENSRNOG 00000006641	GGAACGTCAGCTATGACCA	TCGATCCGACATTCCGAACA
Th	ENSRNOG00000020410	TGTCACGTCCCCAAGGTTCA	TGTACCCCTCAGGGAGAAGAG

### ELISA

Rats were immediately decapitated after the EMP or sham irradiation and prefrontal cortex and hippocampus was removed in ice. The tissue were centrifuged at 4 °C, 3000*g* for 15 min, The levels of 5-HT, DA, epinephrine and NE were measured by ELISA kits (Elabscience, Wuhan, China) according to the manufacturer’s instruction [32].

### Statistical analysis

Data are expressed as mean ± SEM. Unpaired t-test with Welch's correction was performed for comparison between two groups. The distribution of DELs in the chromosome was drawn by the RIdeogram tool. The value of *P* < 0.05 was regarded as a statistical difference.

## Supplementary Information

**Additional file 1.** Differentially expressed lncRNAs between the EMP and the sham group.

**Additional file 2.** Differentially expressed mRNAs between the EMP and the sham group.

**Additional file 3.** The detailed information of the differentially expressed lncRNA-mRNA pairs.

## Data Availability

The data that support the findings of this study are available from the corresponding author upon reasonable request.
